# Block strength training based on age-related functional consequences in older women

**DOI:** 10.1371/journal.pone.0323501

**Published:** 2025-05-30

**Authors:** Emilio Jofré-Saldía, Raúl Ricardo Festa, Álvaro Villalobos-Gorigoitía, Carlos Jorquera-Aguilera, Álvaro Huerta Ojeda, Jorge Cancino-López, Gemma María Gea-García

**Affiliations:** 1 Facultad de Ciencias de la Rehabilitación y Calidad de Vida, Universidad San Sebastián, Región Metropolina, Chile; 2 Sports Performance Research, Rosario, Santa Fe, Argentina; 3 Facultad de Educación y Ciencias Sociales, Instituto del Deporte y Bienestar, Universidad Andres Bello, Santiago, Chile; 4 Facultad de Ciencias, Escuela de Nutrición y Dietética, Universidad Mayor, Santiago, Chile; 5 Núcleo de Investigación en Salud, Actividad Física y Deporte ISAFYD, Universidad de Las Américas, Viña del Mar, Chile; 6 Exercise Physiology and Metabolism Laboratory, Escuela de Kinesiología, Universidad Finis Terrae, Santiago, Chile; 7 Departamento de Educación Física y Deporte- Facultad de Ciencias de la Educación, Universidad de Sevilla, Sevilla, España; Università degli Studi di Milano: Universita degli Studi di Milano, ITALY

## Abstract

Strength training is a form of healthy ageing in older women. Although recommendations currently exist, some are very broad or fail to cover the needs of aging. Therefore, the purpose of this study was to analyze the effects of blocks strength training based on age-related functional consequences on functional performance in older adult women. 82 community-dwelling older women (70.17 ± 6.04 y) were randomly assigned to either experimental (n = 40) or control (n = 42) group. Experimental group performed a Block Strength Training (BST) program based on strength, power, and muscular endurance, and different level of effort for 9 weeks, and control maintained daily routine with physical activity recommendations. Functional performance was assessed using absolute handgrip strength [AHS], timed up and go [TUG], two-minutes step test [2MST], five times stand-to-sit test [5-SST], 6-m walking speed test [6-WS] pre-post intervention. Statistical analyses were performed using two-way ANOVA (Time*Group) and effect size (partial eta-squared, **ŋ*P2*) with a significance level of *p* < 0.05. BST improved functional performance in the protocols of AHS (21.51 vs. 23.07-kg; + 7%), TUG (8.22 vs. 7.29-sec; + 11%), 2MST (78.76 vs. 97.18-steps; + 23%), 5-SST (12.68 vs. 9.43-sec; + 26%), and 6-WS (1.16 vs. 1.36- m·s^-1^; + 17%) compared to control (19.31 vs. 19.66-kg; 8.94 vs. 9.26-sec; 62.68 vs. 63.73-steps; 13.99 vs. 14.25-sec; 1.06 vs. 1.06-m·s^-1^, respectively) in a Time*Group interaction effect (*p* < 0.01; **ŋ*P2*^* *^> 0.11). This BST is effective in improving overall functional performance and thus reducing the risk of physical frailty in community-dwelling older women. These findings strengthen the approach to exercise programming over recommendations, moving toward effective precision dosing for older adults.

## Introduction

Sarcopenia criteria of low muscle mass and function independently predispose to loss of projected physical independence in older adults, but low muscle function has the greatest impact [1]. Sarcopenia is closely linked to fragility, which is an important clinical syndrome of age-related decline in physiologic reserve and increased vulnerability, which is more prevalent in older women [2]. Physical frailty is well exemplified by the Fried phenotype [3] as it is operationalized by features related to functional performance. A multifactorial approach such as GERITRICS 5Ms (mind, mobility, medications, multicomplexity and most importantly) has been promoted to address problems linked to aging [4]; however, this is a broad approach where health problems are already present.

A safe and effective way to avoid falling into severe sarcopenia and thus into physical frailty is resistance or strength training, particularly in older women [5]. In this sense, periodized strength training is more effective than non-periodized [6,7] with blocks programming being a periodization format particularly applied in competitive sports, which is understood as training cycles of highly concentrated specialized workloads that facilitates each mesocycle-block employs its proper combination of training load and exploits the appropriate mode of biological adaptation [8]. Considering this, an interesting strategy for older adults could be applied blocks programming based on the functional consequences of age-related sarcopenia (i.e., ↓ strength; ↓ power; ↓ muscular endurance) [9] with the purpose of improving the functional performance (or daily tasks), and thus inducing effects that move them away from the Fried phenotype for diagnosing frailty (i.e., weakness, poor endurance, slow walking) [3]; something of particular interest for older women given its prevalence [2], which can be ~ 9% compared to ~ 5% in men [10]. Given its specialization, a block program based on the functional consequences of age-related sarcopenia could promote better results than traditional and more general recommendations in older adults. In this sense, we have recently shown how Block Strength Training (BST) based on these consequences of aging improves functional autonomy and quality of life in community-dwelling older women [11], but the program was applied to a medium-sized older women group and not compared with any other intervention. In addition, although functional performance was assessed with some tests, key factors of the functional consequences of sarcopenia such as muscular endurance [9] were not assessed. In contrast, Moreno-Mateos et al. found minors effects on functional performance of BST in older adults, but the block programming had a more traditional and general methodology [12].

In contrast to the contemporary approach [13], current strength training recommendations for older adults maintain a traditional approach by applying a fixed number of repetition-range associated with percentages of one-repetition maximum (i.e., %1RM), although they suggest that these repetition-range should avoid reaching failure [14]. This is crucial due to performing repetitions until failure does not provide further neuromuscular performance gains in older adults [15]; in addition, repetitions until failure also implies higher rating of perceived exertion (RPE) and discomfort/pain than not to failure at a given load [16], which is an aspect to consider mainly in older adults’ populations. In this sense, manipulating the number of repetitions in each training set with respect to the maximum has serious implications on the level of effort due to the relationship between mechanical and metabolic fatigue as the repetitions progress [17]. In support of this approach, applying high to maximum percentage of reps (%rep) with moderate to high loads has been shown to impose high RPE values and unpleasant sensations in multi-joint strength exercises [18,19]. According to the low adherence to strength training recommendations in older adults [20], a program based on avoiding maximal perceptions of effort as unpleasant feelings could potentially improve it. Nevertheless, despite emerging evidence of strength training not to failure in older adults [15,21] and our recent suggestion to program based on the level of effort in the general population [22] little is known about the application of different level of effort (not to failure) within a strength training program, particularly in older adults.

Therefore, the purpose of the present study was to assess the effects of BST programmed according to age-related functional consequences (i.e., focus on strength, power and muscular endurance) and different level of effort (not to failure) on functional performance in older women. We hypothesize that this BST program could have positive effects on these aspects that concern the scientific community.

## Methods

### Participants

Eighty-two community-dwelling older women (70.17 ± 6.04 years) participated in this study. At the time of the interventions, they lived in a rural area and had not participated in any exercise program in the past 6 months. The participants were randomly distributed through the use of specific software (https://www.randomizer.org) into two groups, identified as the experimental (n = 40) and control (n = 42) group. In order to assure the subjects are not limited by the training protocol, the following exclusion criteria were applied: i) impossibility of moving from one point to another without personal or technical assistance; ii) presence of muscle/joint injuries or fractures that occurred in the last three months; iii) terminal illnesses; iv) cardiovascular disease; v) dementia, depression or Alzheimer. After randomization, the similar descriptive characteristics between groups (see Table 1 in the results section), and that they had no more than 2 functional performance tests within the risk cut-off points [23–27] (baseline functional performance; see Table 2 in results section) were used to control the stratification between groups to avoid bias. All participants gave written informed consent to participate in the present study. Calculations to establish the sample size were performed with G*Power 3.1.9.4 software [28]. The calculation established a total sample size of 67 subjects to yield a statistical power of 0.80 with an α = 0.05 and a moderate to large effect size (f = 0.35) according to the statistical method applied in the present study. To avoid possible dropouts or deletion of recorded data due to detection of an abnormal response or dropout, we decided to recruit a larger number of participants (n = 95). Five of the women did not meet the inclusion criteria. Therefore, the groups were randomly and equally distributed (https://www.randomizer.org) into two groups of 45 participants, but the participants were not informed which group they had been assigned to until the interventions began, nor of the potential effects of any of these. After the 9 weeks of intervention, these groups had losses unrelated to the program or intervention (e.g., logistics, moving city), resulting in a final total of 82 participants. Thus, the final sample of the experimental and control groups consisted of 40 and 42 participants, respectively, where experimental group reported no adverse effects related to BST. This research was approved by the Research Ethical-Scientific Committee of Universidad Católica San Antonio de Murcia (code: CE101801). The present study was carried out following the ethical principles established in the Declaration of Helsinki and Ethical Standards for Exercise and Sport Science [29].

**Table 1:** Descriptive characteristics of the experimental and control groups.

	EG	CG	*p-value*	*d*
	M ± SD (n = 40)	M ± SD (n = 42)		
Age (years)	69.08 ± 6.07	71.21 ± 5.90	0.109	-0.358
Height (cm)	152.35 ± 5.10	151.64 ± 8.49	0.650	0.101
Body mass (kg)	71.53 ± 12.82	70.35 ± 13.64	0.688	0.089
BMI (kg/m^2^)	30.67 ± 4.31	30.36 ± 5.10	0.772	0.064
Waist circumference (cm)	92.35 ± 11.05	94.76 ± 11.33	0.332	-0.216
Fat mass (%)	45.98 ± 4.78	45.93 ± 7.35	0.973	-0.008
Muscle mass (%)	22.71 ± 2.50	21.99 ± 2.47	0.207	0.292

EG = experimental group; CG = control group; BMI = Body mass index

*d* = Cohen’s d

**Table 2:** Functional performance before (pre) and after (post) Block Strength Training in each group.

	EG (n = 40)								CG (n = 42)								Post Intervention (n = 82)			
	Pre		Post		Δ (Pre_Post)		*p-value*	*ES*	Pre		Post		Δ (Pre_Post)		*p-value*	*ES*	Δ (CG_EG)		*p-value*	*ES*
	M±	SD	M±	SD	M±	SD			M±	SD	M±	SD	M±	SD			M±	SD		
AHS (kg)	21.51±	4.82	23.07±	4.85	-1.56±	-0.03	0.001**	0.16	19.31±	5.26	19.66±	5.10	-0.35±	0.16	0.375	0.01	-3.41±	0.25	0.003**	0.11
TUG (sec)	8.22±	1.87	7.29±	1.63	0.94±	0.23	0.001**	0.31	8.94±	1.77	9.26±	1.80	-0.32±	-0.04	0.054	0.05	1.97±	0.18	0.001**	0.25
2MST (steps)	78.76±	21.91	97.18±	12.74	-18.41±	9.17	0.001**	0.36	62.68±	26.87	63.73±	24.47	-1.05±	2.40	0.802	0.00	-33.45±	11.73	0.001**	0.46
5-SST (sec)	12.68±	4.47	9.43±	3.46	3.24±	1.01	0.001**	0.45	13.99±	3.90	14.25±	3.92	-0.26±	-0.02	0.502	0.01	4.82±	0.45	0.001**	0.30
6-WS (m·s^-1^)	1.16±	0.31	1.36±	0.30	-0.66±	-0.01	0.001**	0.28	1.06±	0.29	1.06±	0.28	0.18±	0.01	0.936	0.00	-0.30±	-0.02	0.001**	0.22

EG: experimental group; CG: control group; Δ = difference; AHS = absolute handgrip strength; TUG = timed up and go; 2MST = two-minutes step test; 5-SST = five times stand-to-sit test; 6-WS = 6-m walking speed test; ** = statistical difference; ES = effect size.

### Procedures

#### General design.

Subjects were recruited from June 3 to July 26, 2019. Prior to the interventions, anthropometric and estimated body composition assessments were carried out to characterize the already randomized groups. Following two previous familiarization sessions, functional performance was evaluated before and after the training program in both groups through testing protocols (see measurements section). These tests were carried out in an adapted room in a center for older adults between 8:00 and 12:00. To ensure the impartiality of the testing process, the evaluator did not know which group each participant belonged. All participants were asked to maintain their daily routines, usual eating habits, and not take part in any other training programs. Consequently, a BST was applied to experimental group; and control was advised to perform at least 150 min of aerobic physical activity and two muscle strengthening sessions per week according to current WHO recommendations [30] at the beginning of each block through self-care promotion meetings. All resistance training sessions were led by an experienced physical education teacher in training older adults and supervised by the researchers.

#### Block strength training.

**Fig 1:**
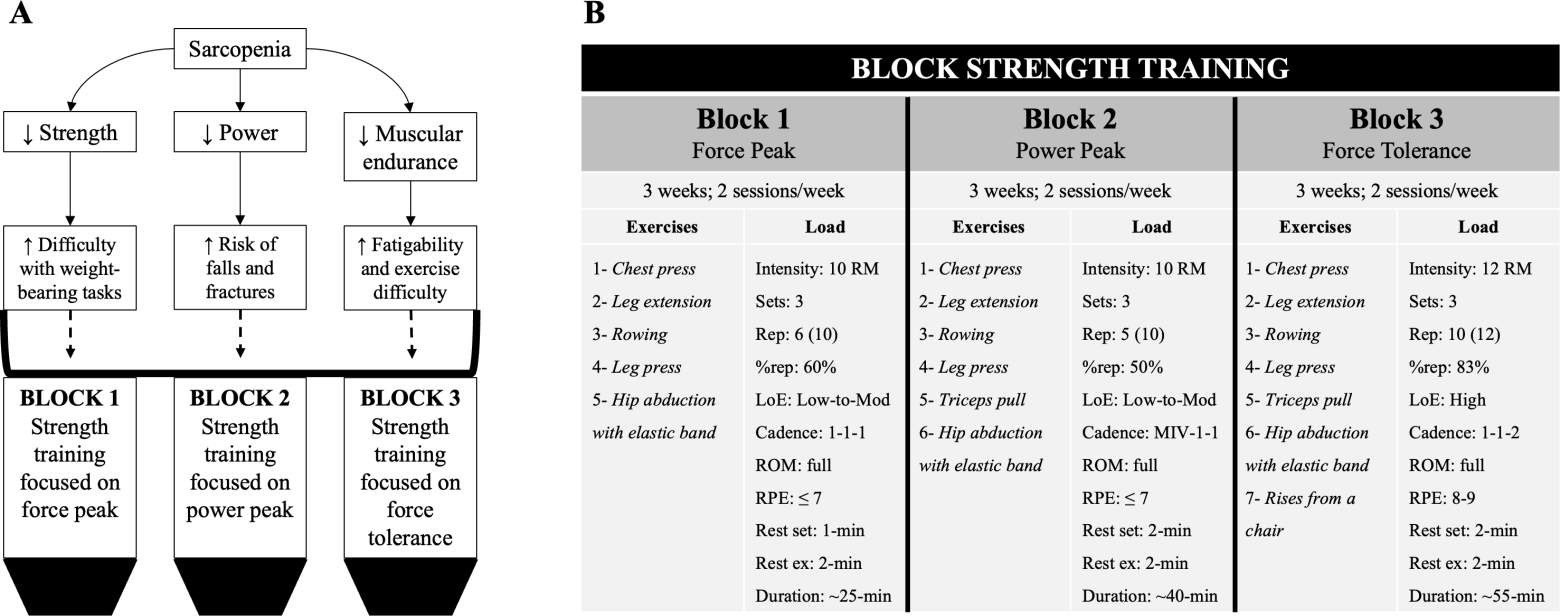
Block Strength Training (BST). Panel A shows the model of the functional consequences of age-related sarcopenia proposed by Hunter et al. [9] (top) and its link to the blocks of the strength training program (bottom). Panel B describes the BST in detail. C: Cadence (seconds) during concentric, isometric, and eccentric phases; LoE: level of effort; MIV: maximal intended velocity; RPE: rating of perceived exertion; Rep: Repetitions (i.e., repetitions performed (maximum repetitions possible)); %rep: percentage of repetitions with respect to a maximum; Rest ex: Rest between exercises; ROM: Range of motion.

The program lasted for 9 weeks; it was subdivided into 3 blocks of 3 weeks each as described in Fig 1. The blocks were designed according to the model of functional consequences of age-related sarcopenia (i.e., ↓ strength; ↓ power; ↓ muscular endurance) proposed by Hunter et al [9]. In this sense, each block used a specific %rep to guide the effects based on the potential velocity loss [31,32]. BST was applied twice a week in non-consecutive morning sessions and in groups of 10–15 participants. Every training session consisted of 10-min of warm-up (joint mobility), ~ 25–55-min of strength exercises and 5-min of cool-down (stretching). Progressive workload between blocks was addressed by the length of the main section of each session (work + rest), the number and time under tension (cadence) of the exercises, and the number of repetitions applied (set x reps). The professional in charge of leading the sessions recorded attendance in the experimental group in order to indirectly control adherence to BST. In this sense, meetings were held with the control group at the beginning of each block to promote the physical activity recommendations already mentioned, and to reinforce the idea of not participating in other exercise programs regularly. According to the fact that traditional multi-joint exercises of upper and lower limbs typically reach between ~10–20 reps with ~65–75% of 1RM [31,33], external loads were individualized using the number repetition maximum test [34] in the main exercises (e.g., leg press; chest press) every 6 sessions (i.e., 10RM and 12RM, respectively); which were performed on exercise machines (Rojas Sport®, Chile). To adjust the level of effort from low to high during the program, both the RPE scale 0–10 (i.e., ≤ 7 and 8–9) and %rep (i.e., 50–60% and 83%) were applied [18,31]. Both estimated relative intensity and cadence were programmed in line with recent power training recommendations for older adults [35], and the other main prescription variables followed to the basic guide proposed by Bavaresco Gambassi et al. [36]. We have suggested RPE to support %rep control because it is an effective tool both at controlled speed [37] and at maximum intended velocity [18]. For the accessory exercises (e.g., hip adduction; rising from a chair), elastic bands of different tension (Fullfit®, Chile) and medicine balls of different weight (HWM®, Chile) were used to apply the same approach of %rep of number repetition maximum test. Level of effort based on RPE and %rep was explained at the beginning of the intervention and were reviewed in each session. For pedagogical reasons, the cadence of repetitions was controlled in the BST (KDM-2 digital metronome, Korg Inc., Japan), which also directed the desired effects in each training block (see Fig 1).

#### Body composition.

To evaluate and estimated this variable, the electrical impedance measurement technique was used (OMRON HBF-514®, OMRON Healthcare, Inc., USA). In addition, waist circumference assessment was performed using an anthropometric tape which has an accuracy of 0.1-cm (SECA S201®, Germany).

#### Handgrip strength.

Participants performed the test sitting on a chair without armrests with the elbow flexed at 90°, where at the signal of the evaluator they had to press the device for 3-s; two trials were performed with the dominant limb (1 minute of rest) and the highest value was recorded. In older women, an absolute handgrip strength (AHS) of 16–20-kg is classified as intermediate weakness and < 16-kg as weak [23]. A manual device was used to assess grip strength (CAMRY EH101®, USA).

#### Timed up and go.

The Timed Up and Go (TUG) test was used to evaluate the specific mobility and functionality of the lower limbs. The time that a person takes to stand up from a chair, walk 3-m, turn to cone, walk back to the chair, and sit down was recorded. In this test, having a time of > 12-sec has a substantial impact on the probability of fracture risk in older women [24]. Time was controlled using a digital stopwatch (Polar® V800, Finland).

#### Two-minutes step test.

To evaluate endurance performance, the two-minutes step test (i.e., 2MST) was used, counting the number of times the right knee reached the height between the kneecap and the anterior superior iliac spine. In this protocol, ≤ 60 steps accurately identifying functional impairment in older adults [25]. The number of steps was controlled by an evaluator.

#### Five times stand-to-sit test.

To screen for muscle weakness in lower limbs, the action of getting up and sitting down from an armless chair was performed five times (5-SST) as quickly as possible, with their arms crossed across their chests. A performance of > 11.64-sec on this test may identify reduced muscle strength in community-dwelling older women [26].

#### Walking speed.

Walking speed covering a distance of 6-m (6-WS) was evaluated to determine performance speed. In this context, walking speed < 0.8 m·s^-1^ is diagnosis of frailty in the primary care [27]. A digital stopwatch (Polar® V800, Finland) was used to record the time they took to complete the distance.

### Statistical analysis

Data are presented as mean (M) and standard deviation (SD). Normality and homogeneity of the sample were checked by using the Kolmogorov-Smirnov and Levene tests respectively (*p* > 0.05). To compare the descriptive anthropometric and body composition characteristics of the groups, a student’s t test or Mann-Whitney test was performed. Two-way repeated measures analysis of variance (Two-way ANOVA) was applied with TIME and GROUP as factors to analyze the possible effects on functional performance. Bonferroni post-hoc test was applied to explore possible differences between each of the two conditions. Friedman repeated measures test was applied with post hoc through Wilcoxon in non-normal distribution variables. The effect size (ES) was estimated by calculating Cohen’s *d* [<0.2 (small); > 0.5 (medium) and > 0.8 (large) effect] and the partial eta-squared (**ŋ*P2*) [<0.01 (small); > 0.06 (medium) and > 0.14 (large) effect], respectively. A significance level of *p* < 0.05 was accepted for all statistical comparisons. Calculations were performed using the statistical programs SPSS Statistics for Windows, version 25.0 (IBM Corp., USA).

## Results

At the time of the study, the 82 participants were 70.17 ± 6.04 years old, while anthropometric assessments showed a height of 151.98 ± 7.01 cm, a body mass of 70.93 ± 13.18 kg, a body mass index of 30.51 ± 4.70 kg/m^2^, and a waist circumference of 93.58 ± 11.19 cm. Furthermore, the percentage of fat mass was 45.95 ± 6.19% and that of muscle mass was 22.34 ± 2.50%. The similar characteristics of each group are shown in Table 1. There were no dropouts in any of the groups from the beginning to the end of the interventions.

### Handgrip strength.

Two-way ANOVA revealed a statistically significant interaction between the effects of TIME and GROUP on AHS (F_1_ = 9.52, *p* = 0.01, **ŋ*P*2 = 0.11). Simple main effects analysis showed a statistically significant pre vs. post intervention intra-group effect on AHS (F_1_ = 4.65, *p* = 0.03, **ŋ*P2* = 0.06). Simple main effects analysis showed a statistically significant post intervention inter-group effect on AHS (F_1_ = 6.79, *p *= 0.01, **ŋ*P2* = 0.08). The results are shown in Table 2.

### Timed up and go.

Two-way ANOVA revealed a statistically significant interaction between the effects of TIME and GROUP on TUG (F_1_ = 24.48, *p* = 0.01, **ŋ*P2* = 0.25). Simple main effects analysis showed a statistically significant pre vs. post intervention intra-group effect on TUG (F_1_ = 29.54, *p* = 0.01, **ŋ*P2* = 0.29). Simple main effects analysis showed a statistically significant post intervention inter-group effect on TUG (F_1_ = 11.72, *p* = 0.01, **ŋ*P2* = 0.14). The results are shown in Table 2.

### Two-minutes step test.

Two-way ANOVA revealed a statistically significant interaction between the effects of TIME and GROUP on 2MST (F_1_ = 45.02, *p* = 0.01, **ŋ*P2* = 0.46). Simple main effects analysis showed a statistically significant pre vs. post intervention intra-group effect on 2MST (F_1_ = 10.60, *p* = 0.01, **ŋ*P2* = 0.16). Simple main effects analysis showed a statistically significant post intervention inter-group effect on 2MST (F_1_ = 22.87, *p* = 0.01, **ŋ*P2* = 0.30). The results are shown in Table 2.

### Five times stand-to-sit test.

Two-way ANOVA revealed a statistically significant interaction between the effects of TIME and GROUP on 5-SST (F_1_ = 32.35, *p *= 0.01, **ŋ*P2 *= 0.30). Simple main effects analysis showed a statistically significant pre vs. post intervention intra-group effect on 5-SST (F_1_ = 38.66, *p* = 0.01, **ŋ*P2* = 0.34). Simple main effects analysis showed a statistically significant post intervention inter-group effect on 5-SST (F_1_ = 12.83, *p* = 0.01, **ŋ*P2* = 0.15). The results are shown in Table 2.

### Walking speed.

Two-way ANOVA revealed a statistically significant interaction between the effects of TIME and GROUP on 6-WS (F_1_ = 22.04, *p* = 0.01, **ŋ*P2* = 0.22). Simple main effects analysis showed a statistically significant pre vs. post intervention intra-group effect on 6-WS (F_1_ = 16.69, *p* = 0.01, **ŋ*P2* = 0.17). Simple main effects analysis showed a statistically significant post intervention inter-group effect on 6-WS (F_1_ = 10.93, *p *= 0.01, **ŋ*P2* = 0.12). The results are shown in Table 2.

**Fig 2:**
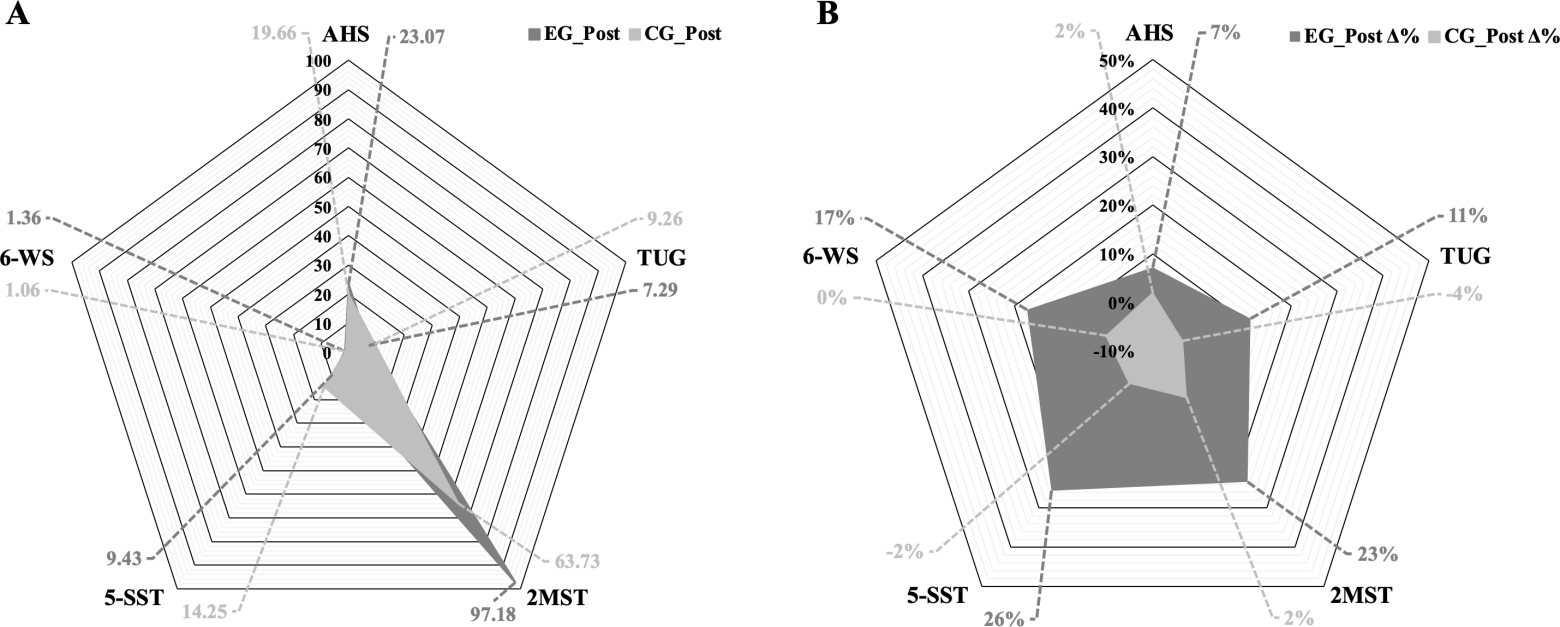
Functional performance profile of the experimental group and the control group. Panel A shows the absolute post-intervention profile in both groups. Panel B shows the relative profile post-intervention (percentage change, or Δ%) in both groups. AHS = absolute handgrip strength; EG: experimental group; CG: control group; Δ% = percentage change; TUG = timed up and go; 2MST = two-minutes step test; 5-SST = five times stand-to-sit test; 6-WS = 6-m walking speed test.

Additionally, Fig 2 shows the profiles of absolute functional performance (panel A), and the relative changes in functional performance (panel B) of each group 9 weeks of intervention.

## Discussion

The results of the present study reveal this novel and specific BST based on age-related functional consequences could be effective in improving the general functional performance in older women compared to physical activity recommendations. Regarding the most notable improvements, these were observed in endurance/tolerance (i.e., 2MST), lower limb muscle strength (i.e., 5-SST), and walking speed performance (i.e., 6-WS), capabilities typically used in daily tasks. Therefore, these results transform this BST into a frailty-prevention program, and strengthens the exercise programming approach rather than recommendations.

### Strength training programming

Currently, the benefits of resistance exercise are being recognized and is not the exclusive domain of power- or strength-training athletes; these are a form of exercise that leads to good health and healthy aging [38], which extend far beyond muscle hypertrophy and the requirement to lift heavy weights [39]. The current recommendations for strength training in older adults described by Izquierdo et al. and Fragala et al. maintain a traditional approach based on a fixed number of repetitions or ranges linked to %1RM [14,40]. Although the application of a range of repetitions that avoids reaching muscular failure is proposed [14], it is not addressed from the perspective of level of effort like the present study, which can generate different level of effort at a given load due to the interindividual variability of repetitions [31,33] affecting the desired effects of the program [32].

Previous studies of phased or block strength training indicate that not applying the maximum number of repetitions provides favorable conditions to improve both strength and muscle power, and performing sets to maximum %reps or failure result in greater gains in muscular endurance in young adult athletes [41], something that could be explained by the different metabolic alterations that each methodology causes [42]. It is interesting to note that in the present study, even muscular endurance (i.e., 2MST) improved without incurring training to maximum number of repetitions or to failure, which is important because the latter may be associated with greater discomfort and displeasure [16,19] as well as higher metabolic and mechanical fatigue [17], and potentially negatively affect long-term exercise adherence in older adults; however, this should be studied [43].

The blocks applied in the present study were not designed arbitrarily, since they are in line not only with the functional consequences of sarcopenia [9] but also with the proposed phases of neuronal and muscular adaptation proposed by Digby G. Sale [44], which could explain the overall effects on functional performance given that this BST allows for rational sequencing of specialized mesocycle-blocks that employ exploitation and superposition of residual training effects, facilitated by its proper combination of training loads suitable to various modes of biological adaptation [8]. In short, the main objective of our BST is to address the age-related decline in motor performance and the within- and between-subject variability of several of its aspects (e.g., force, velocity, and fatigability) that increase with advanced aging [45]. Therefore, this BTS could be considered as a frailty-prevention program since the effects move participants away from the Fried phenotype for frailty [3]. Furthermore, given the effects that this BST generates on functional performance compared to physical activity recommendations [30], the exercise programming approach to achieve the desired results is strengthened. In addition, a recent meta-analysis found that strength training had significant positive effects on physical factors associated with frailty (i.e., handgrip strength, gait speed), with few exceptions (i.e., TUG, chair stand time tests) [46]. Our results are not only in line with those of the meta-analysis; they even support favorable evidence for the exceptions (i.e., TUG, chair stand time tests) [46].

### Effects of BST on functional performance

Block training is traditionally used in competitive sport [8], but little is known about its application in the general population, particularly in older women. In this regard, Galdames Maliqueo et al. have recently demonstrated the effectiveness of a similar BST on functional autonomy tests belonging to the Latin American Group for Maturity protocol on a group of 18 community-dwelling older women [11]; specifically, finding improvements in walking speed and the 5-SST in line with our results. On the other hand, the outcomes of Moreno-Mateos et al. suggest caution with the effectiveness of a BST relative to a non-periodized multicomponent intervention in older adults, given that the BST only improve TUG, and the non-periodized intervention only the two-minute staircase test, without causing changes in walk speed, AHS and 5-SST [12]. We think that the lack of comparison between groups due to non-homogeneity, as well as the less specialized and general programming based on volume (>15 repetitions at low speed), intensity (<15 repetitions at high speed) and exercises similar to those of the evaluations [12], could explain the conclusions different from ours.

The BST of the present study not only shows to be efficient in improving overall functional performance compared to physical activity recommendations [30], but shows to be a time-effective program. For example, the number of reps per session in each block was 90–210, respectively; compared to 150–252 reps if the maximum number of reps or failure were applied. This indicates that a lower training volume than possible (i.e., lower level of effort) improves functional performance in older women, demonstrating that training to muscle failure is not a prerequisite for this. In this sense, the evidence is consistent in pointing out that strength training with moderate intra-set velocity loss (i.e., moderate %rep) should be chosen to optimize neuromuscular adaptations [47,48], showing that low %rep optimizes gains in older adults [15]. For instance, Cadore et al. found improvements in the 5-SST and relative jump power, but not in the TUG, after 12 weeks of concurrent strength training to failure and 50%rep in older adults [43]. Similar results were found by Bergamasco et al. after low-load no to failure strength training which improved 5-SST and habitual gait speed, but not maximal walking speed and TUG in 67-year-old subjects [49]. Marques et al. in a recent study showed that a low-volume strength training based on intra-set velocity loss induces improvements in strength and functional performance in older adults, indicating that a 10% velocity loss is more efficient as it requires less volume or total repetitions than a 20%, but the latter should be considered for some improvements [50]. All of these studies support lower volume (i.e., lower level of effort) programming as a strategy to optimize outcomes in older adults, thereby avoiding exposure to higher cardiovascular and metabolic stress from higher-volume training [51,52]. However, to our knowledge, the present study is novel because it applies a program based on different level of effort, achieving improvements in a broad spectrum of functional performance tests.

The improvements in the functional performance of the present study should not only be highlighted at relative (i.e., ~ 7–26%) and statistical level (*p* < 0.01; **ŋ*P2* ≥ 0.11), but also at a strictly functional level. After the BST, the experimental group mean moves away from the intermediate weakness classification for AHS, where control falls within it (i.e., 16–20-kg) [23]. In the TUG, the experimental group improves its performance, reduces the risk of fracture probability compared to the control, which after the intervention remains closer to the cut-off point where the risk increases (>12-sec) [24]. On the other hand, although the experimental group begins the BST with good values in the 2MST, they improve even more and move away from the lower performance that identifies functional impairment in older adults (i.e., ≤ 60 steps) [25], which persists in the control. The 5-SST is the test with the most relevant effect, due to prior to the interventions both groups could be classified as older women with reduced muscular strength (i.e., > 11.64-sec) [26], but after the program the experimental improved its performance to extent that it moved away from this classification compared to control. This result could be explained by the programmed neuromuscular approach of the first 2 blocks and the biomechanical specificity of exercise included in the third block according to daily needs (i.e., rises from a chair). Finally, although in the 6-WS both groups are far from the diagnosis of frailty (i.e., < 0.8 m·s^-1^) [27] prior to the interventions, the improvements in the experimental group move it even further away from it. The positive general functional performance prior to the interventions could be associated with the lifestyle of the place of residence at the time of the study [53]. However, we believe that the post-intervention results in the control group suggest that physical activity recommendations are not sufficient, and structured exercise programs are required.

### Future research and limitations

Finally, it is worth highlighting some considerations. Future studies should apply the BST of the present study, but assign a broader spectrum of loads (i.e., light to heavy) and include men, as well as analyze program response by age range, with the aim of expanding and consolidating the results. During the development, some limitations were observed that could impact the interpretation and generalization of the results: a) the sample studied was composed exclusively of older women from a rural area; this situation limits the applicability of the findings to other populations; b) the absence of a long-term follow-up prevents evidence of whether the observed adaptations (i.e., functional performance) are permanent over time; c) nutrition was not controlled during the intervention, a variable that could have influenced the results; d) finally, the design did not include an experimental group with a conventional training method (e.g., training to failure or maximum %rep); therefore, it could not be determined whether the effect achieved with the BST is superior to other strength programs.

## Conclusions

In conclusion, a BST program based on age-related functional consequences and low to high level of effort not to failure is an effective mean to improve overall functional performance in community-dwelling older women, potentially maintaining independence by improving typical daily tasks and reducing the risk of becoming physically frail. These findings strengthen the approach to exercise programming over recommendations, moving toward effective precision dosing for older adults.

## Practical applications

Strength training should be thought of far beyond muscle hypertrophy and lift heavy weights [39], as it is currently considered to lead to good health and healthy aging [38]. This novel BST based on age-related functional consequences is a program toward this consideration, as its programming focuses on age-specific needs that result in potential improvements in daily tasks for older women, and therefore in their quality of life. In addition, this BST shows that training to failure is not a prerequisite for achieving outcomes in older adults, as other studies have already shown [43,49,54]. In this sense and according to the low adherence to strength training recommendations in older adults [20], we believe that this BST is promising in this topic, particularly because it is based on level of effort, although we are aware that more studies are needed that assess adherence beyond program attendance.

## Supporting information

S1 Data BST_Data_Set. (XLSX)
